# Geographic prediction of tuberculosis clusters in Fukuoka, Japan, using the space-time scan statistic

**DOI:** 10.1186/1471-2334-7-26

**Published:** 2007-04-11

**Authors:** Daisuke Onozuka, Akihito Hagihara

**Affiliations:** 1 Department of Information Science, Fukuoka Institute of Health and Environmental Sciences, 39 Mukaizano, Dazaifu, Fukuoka 818-0135, Japan; 2 Department of Health Services Management and Policy, Kyushu University Graduate School of Medicine, 3-1-1 Maidashi, Higashi-ku, Fukuoka 812-8582, Japan

## Abstract

**Background:**

Tuberculosis (TB) has reemerged as a global public health epidemic in recent years. Although evaluating local disease clusters leads to effective prevention and control of TB, there are few, if any, spatiotemporal comparisons for epidemic diseases.

**Methods:**

TB cases among residents in Fukuoka Prefecture between 1999 and 2004 (n = 9,119) were geocoded at the census tract level (n = 109) based on residence at the time of diagnosis. The spatial and space-time scan statistics were then used to identify clusters of census tracts with elevated proportions of TB cases.

**Results:**

In the purely spatial analyses, the most likely clusters were in the Chikuho coal mining area (in 1999, 2002, 2003, 2004), the Kita-Kyushu industrial area (in 2000), and the Fukuoka urban area (in 2001). In the space-time analysis, the most likely cluster was the Kita-Kyushu industrial area (in 2000). The north part of Fukuoka Prefecture was the most likely to have a cluster with a significantly high occurrence of TB.

**Conclusion:**

The spatial and space-time scan statistics are effective ways of describing circular disease clusters. Since, in reality, infectious diseases might form other cluster types, the effectiveness of the method may be limited under actual practice. The sophistication of the analytical methodology, however, is a topic for future study.

## Background

Tuberculosis (TB) has reemerged as a global public health epidemic in recent years. TB remains a serious public health problem among certain patient populations, and is prevalent in many urban areas. The World Health Organization estimates that approximately nine million individuals will develop active TB disease and more than two million will die from TB [1,2]. The global burden of TB remains enormous, and will likely rank high among public health problems in the coming decades [3-5].

Although TB cases in Japan had decreased in number until 1996, the prevalence suddenly began to increase between 1997 and 1999. After a 4-year rise, the TB prevalence again began to decrease in 2000. Since that time, about 30,000 TB cases have been reported annually. In 2004, 29,736 cases were reported [6] (23.3 cases per 100,000 individuals). The incidence rate of TB is generally higher in metropolitan areas than in rural areas. For example, the incidence in Osaka (61.8) is 5.9 times higher than the incidence in Nagano (10.4), and the incidence in Tokyo (34.7) is 3.3 times higher than in Nagano. For international comparison, the incidence rate in Japan (23.3) is 5.1 times higher than in Sweden (4.6), 4.4 times than in Australia (5.3), and 4.8 times than in the United States (4.9). Therefore, TB remains a major infectious disease in Japan.

The TB incidence in Fukuoka Prefecture, located on Kyushu Island, is higher than the national average, and is 42nd (6th worst) among the country's 47 prefectures. In 2004, 1,295 cases were reported [7] (25.6 cases per 100,000 individuals). Since 2000, the TB incidence in the prefecture has decreased. Population-based studies have reported numerous human factors that are related to genotype clustering in TB, including younger age, nationality, black race, male sex, positive respiratory acid-fast bacilli smear, acquired immunodeficiency syndrome (AIDS), human immunodeficiency virus (HIV) infection, homelessness, drug and alcohol abuse, and failure to identify contacts [8]. We can safely conclude that individuals who live in these fragile conditions generally have an elevated risk of TB infection [9-11]. Until the 1970s, several coal mines operated in Fukuoka Prefecture, and in these coal mining areas, TB was prevalent [12]. The incidence of pneumoconiosis and COPD, which result from the working conditions in coal mines [13-17], has been on the decline since the closure of the coal mines. In addition, environmental control in those areas has been stringent. Consequently, TB, COPD, and pneumoconiosis appear, at least superficially, to have been suppressed. However, the prevalence of TB is higher in the coal mining area than in the rest of Fukuoka Prefecture, although for technical reasons, it was difficult to determine where and when the prevalence was high before the advent of spatial, temporal, and space-time scan statistics.

Public health officials are often required to evaluate local disease cluster alarms. After the case definition is established, officials try to determine whether the cluster occurred by chance. A high number of disease cases might be due to a common elevated risk factor of limited geographical or temporal extension. In this case, a more thorough investigation is warranted to identify risk factors. It is not statistically suitable to simply compare the standardized disease incidence rate within the cluster area and time frame with the rate observed in a larger geographic area and time frame. In this type of comparison, the spatial and temporal boundaries of the cluster are defined from an observed set of cases, which produces a preselection bias in the statistical analysis. Moreover, any geographical region always contains high-rate areas that occur by chance alone. If every cluster of cases found to be statistically significant, according to such a procedure, were to be investigated thoroughly, public health officials would investigate mostly random data.

Spatial, temporal, and space-time scan statistics are now commonly used to detect and evaluate disease clusters [18-21] for a variety of diseases, including cancer [22,23], Creutzfeldt-Jakob disease [24], granulocytic ehrlichiosis [25], sclerosis [26], and diabetes [27]. SaTScan software analyzes spatial, temporal, and space-time data using spatial, temporal, or space-time scan statistics [28-30]. SaTScan™ is a trademark of Martin Kulldorff. The SaTScan™ software was developed under the joint auspices of Martin Kulldorff, the National Cancer Institute and Farzad Mostashari at the New York City Department of Health and Mental Hygiene. The space-time scan statistic is useful for determining which cluster alarms merit further investigation and which clusters are likely to be occurring by chance [31]. In this study, we used this state-of-the-art statistic to identify where and when the prevalence of TB is high in Fukuoka Prefecture. Given the properties of TB, any single case represents a sentinel event warranting public intervention. However, in the case of TB, it is necessary to know which cluster alarms merit further investigation and which clusters are likely to have occurred by chance. In other words, the ability to detect disease outbreaks is important for local and national health departments to minimize morbidity and mortality through timely implementation of disease prevention and control measures. Because the statistic meets these needs completely, results that are effective and practical for public health officials are expected from this study.

## Methods

### Data sources

**Figure 1:**
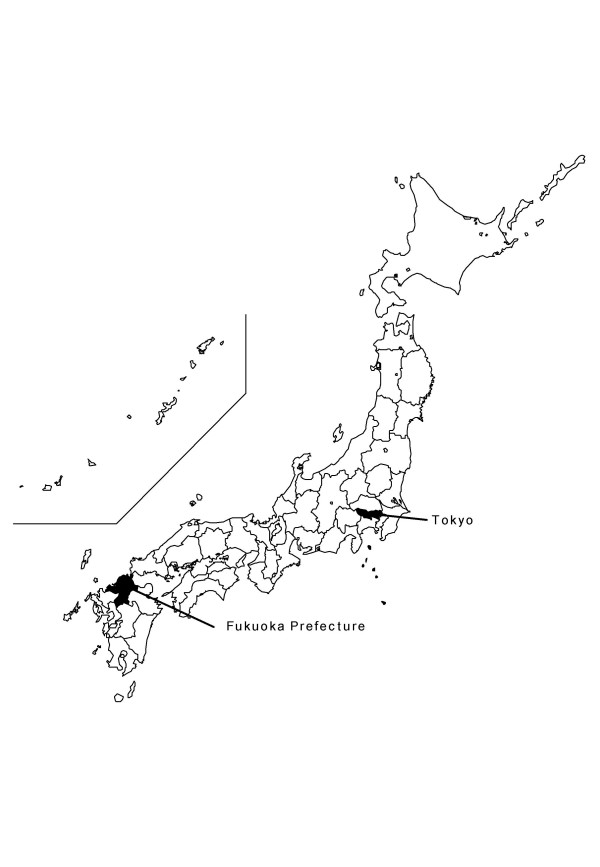
Location of Fukuoka Prefecture on Kyushu, southwest of Tokyo, Japan.

Relevant data for Fukuoka Prefecture, which is southwest of Tokyo, Japan (Fig. 1) were used in the study. Specifically, tests for spatial randomness were applied to six different geographic data sets (Table 1). Demographic data were compiled for each of the 109 Fukuoka Prefecture census tracts from the Japan Census. Data on TB cases in Fukuoka Prefecture were obtained from the TB surveillance system, which monitors TB events among the roughly five million residents in Fukuoka Prefecture. Addresses were matched to towns for 9,119 TB cases from 1999 to 2004. All cases were geocoded to latitude and longitude coordinates. We used 109 different geographic TB data sets for the spatial scan statistic, in each year. Finally, this study using the TB surveillance data was approved by the Fukuoka Prefecture Environmental Health Research Advancement Committee (ethics committee) on December 17, 2004 (Reference Number: 16-1803).

**Table 1:** Summary of the data sets

Data type	Ages	Geographic area	Spatial resolution	Locations (n)	Year	Total cases(n)	Male cases(n)	Female cases(n)
Incidence	All	Fukuoka, Japan	Towns	109	1999	1,845	1,156	689
Incidence	All	Fukuoka, Japan	Towns	109	2000	1,613	1,050	563
Incidence	All	Fukuoka, Japan	Towns	109	2001	1,553	993	560
Incidence	All	Fukuoka, Japan	Towns	109	2002	1,415	899	516
Incidence	All	Fukuoka, Japan	Towns	109	2003	1,398	829	569
Incidence	All	Fukuoka, Japan	Towns	109	2004	1,295	806	489

Source. Fukuoka Prefectural Government: Statistics of TB 2005 in Fukuoka Prefecture.

### Data analysis

The spatial scan statistic was developed to test for geographical clusters and to identify their approximate location [20]. The number of events, *i*.*e*., incident cases, may be assumed to have either a *Poisson *or *Bernoulli *distribution. Depending on the availability of data, the spatial scan statistic can be used for either aggregated data, such as census areas, or with precise geographical coordinates, where each 'census area' contains only one person at risk.

The spatial scan statistic imposes a circular window on the map, and lets the center of the circle move over the area, so that the window includes different sets of neighboring census areas at different positions. If the window contains the centroid of a census area, that entire area is included in the window. For each circle centroid, the radius of the circular window varies from 0 to a maximum radius, so the window never includes more than 50% of the total population at risk. In this way, the circular window is flexible in both location and size. The method creates a very large number of distinct circular windows, each containing a different set of neighboring areas, and each possibly containing a cluster of events [21].

Using the total number of cases in the region, the scan statistic tests the null hypothesis, complete spatial randomness, against the alternative hypothesis, which is that the probability of a case being inside the zone is greater than it than being outside of the zone. Since the scan statistic is likelihood-based, the most likely zones can be selected and tested for statistical significance. Because we are evaluating a huge number of outbreak locations, sizes, and durations, we need to adjust for multiple testing. Since we do not have population-at-risk data, this cannot be achieved via standard methods of scan statistics. Instead, we must create a large number of random permutations on the spatial and temporal attributes of each case in the data set. That is, we shuffle the times and assign them to the original set of case locations, ensuring that both the spatial and temporal marginals remain unchanged. Then, the most likely cluster is calculated for each simulated data set in exactly the same way as for the real data. To estimate distributions for the test statistic, the spatial scan statistic uses the *Monte Carlo *method and assigns cases to individuals in the population randomly, and recalculates the test statistic. Only a small number of all possible zones (potential clusters) are tested, to minimize multiple testing and false positives. In each of the likely clusters, the output includes a listing of geographic subdivisions, numbers of observed and expected cases, population, relative risk (RR), and *p*-value. The scan test imposes a circular zone and calculates a rate within that zone. Areas of below-average risk within that zone will be declared a cluster constituent, provided their low rate is offset by high-rate areas within the circular zone. Thus, the scan statistic is prone to false-positives.

If a purely spatial analysis is performed for an extensive time period, it is more difficult to detect recently emerging clusters. To resolve this problem, a purely spatial analysis based on data from only the last few years can be performed. One problem, however, is determining the appropriate number of years to include. If too few years are analyzed, low-to-moderate excess risk that is present for a considerable length of time could be missed. If too many years are analyzed, the analysis might have insufficient power to detect a very recent high-excess-risk cluster. One solution might be the use of a space-time scan statistic.

Instead of a circular window in two dimensions, the space-time scan statistic uses a cylindrical window in three dimensions. The base of the cylinder represents space (as in the purely spatial scan statistic), and the height represents time. The cylinder is flexible in both its circular geographical base and its starting date, which are independent of one other. As in the purely spatial scan statistic, the likelihood ratio test statistic is constructed using a computational algorithm for calculating the likelihood for each window in three dimensions, rather than in two dimensions.

Spatial scan statistic (SaTScan) was used to identify clusters of census tracts in Fukuoka Prefecture. SaTScan identifies a cluster at any location of any size up to a maximum size, and minimizes the problem of multiple statistical tests. Scanning was also set to search only for areas with high proportions of TB. No geographic overlap was used as a default setting, so secondary clusters would not overlap the most significant cluster. In order to scan for small to large clusters, the maximum cluster size was set to 50% of the total population at risk. To ensure sufficient statistical power, the number of *Monte Carlo *replications was set to 999, and clusters with statistical significance of *p *< 0.05 were reported.

Since the spatial scan statistic (SaTScan) has been widely applied to various diseases, we applied the method, utilizing a circle as the base for the scanning cylinder, to the data for Fukuoka Prefecture. A less geographically compact cluster provides less power to detect the disease. All the clusters detected were approximately circular. In reality, however, infectious diseases might frequently assume other cluster types, and more complicated clusters, such as oval clusters, are more realistic. We were aware of this methodological limitation and sought to exercise caution when interpreting the results.

## Results

Before reporting the results, we refer to the meaning of background risk for TB. Even in a society without known risk factors for TB, some TB cases (*i*.*e*., the expected number) are observed. The TB clusters are calculated based on the expected numbers. Since the absence of background risk does not necessarily indicate the absence of disease, we must again be careful in interpreting the results.

**Figure 2:**
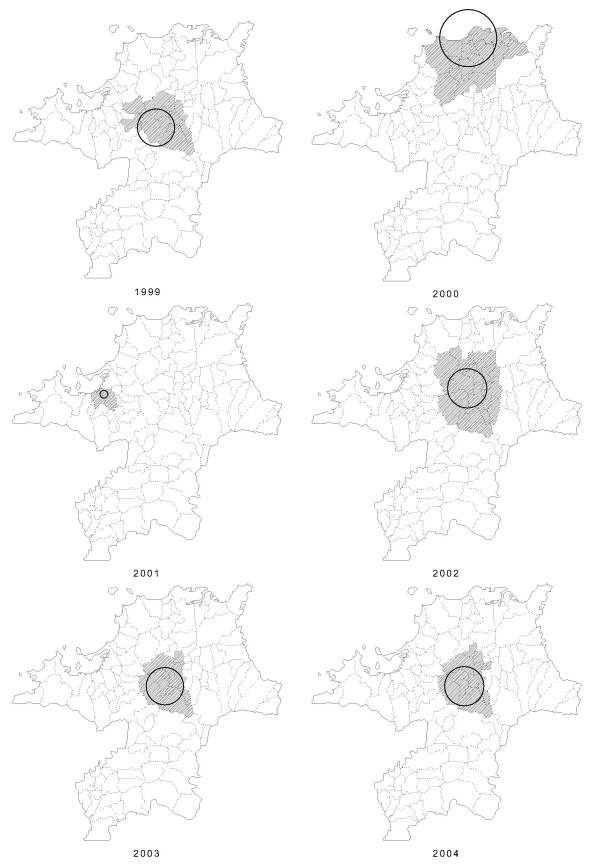
Locations of the detected clusters of TB cases from 1999 to 2004, based on a purely spatial analysis.

**Figure 3:**
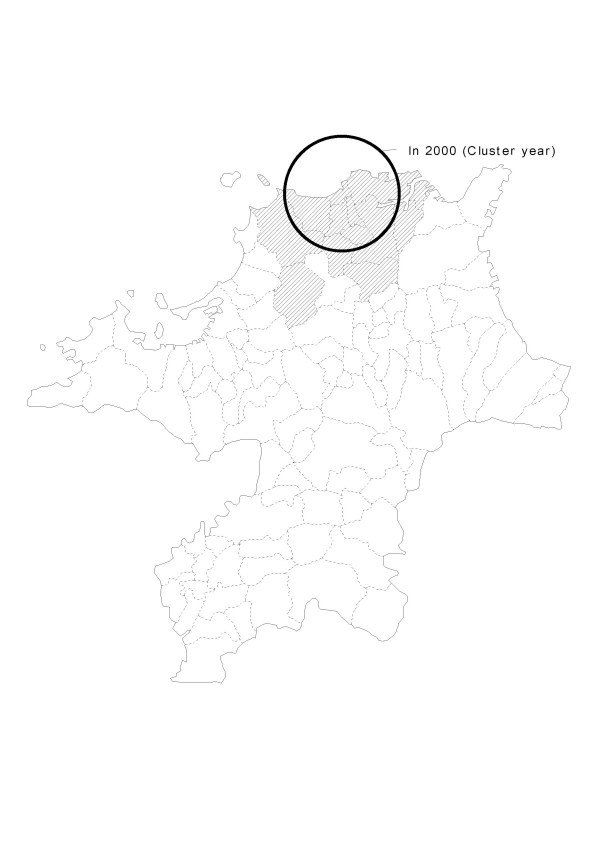
Locations of the detected clusters of TB cases, using historical data from 1999 to 2004, based on the space-time analysis.

The results of the purely spatial analysis of TB data from 1999 to 2004 are shown in Tables 2 and 3, and in Figures 2 and 3. The most likely significant clusters for a high occurrence of TB are listed in Table 2, and depicted on the map in Figure 2. For the purely spatial analyses, the most likely cluster was detected for each year. The most likely cluster had 138 observed cases in 1999, when 96.9 were expected theoretically (RR = 1.46, *p *= 0.013); 437 *vs*. 333.8 cases (RR = 1.42, *p *= 0.001) in 2000; 135 *vs*. 97.6 cases (RR = 1.42, *p *= 0.045) in 2001; 193 *vs*. 117.9 cases (RR = 1.74, *p *= 0.001) in 2002; 106 *vs*. 55.7 cases (RR = 1.98, *p *= 0.001) in 2003; and 100 *vs*. 55.1 cases (RR = 1.88, *p *= 0.002) in 2004. The most likely clusters were the Chikuho coal mining area (in 1999, 2002, 2003, 2004), Kita-Kyushu industrial area (in 2000), and Fukuoka urban areas (in 2001). We also detected several secondary clusters, which were not significant.

Next, we tested the space-time analysis of the TB data from 1999 to 2004. The most likely significant clusters for a high occurrence of TB are listed in Table 3, and depicted on the map in Figure 3. In the 1999–2004 data, the most likely cluster had 354 cases, when 287.8 were theoretically expected (RR = 1.23, *p *= 0.015) in 2000. In the 2000–2004 data, the most likely cluster had 354 cases when 282.1 were theoretically expected (RR = 1.26, *p *= 0.001) in 2000. We also detected several secondary clusters, which were not significant.

**Table 2:** The mostly likely clusters of TB cases detected using the purely spatial analysis

Data Years	Cluster Areas(n)	Observed Cases	Expected Cases	Relative Risk	p-value
1999	11	138	96.9	1.46	0.013
2000	16	437	333.8	1.42	0.001
2001	2	135	97.6	1.42	0.045
2002	20	193	117.9	1.74	0.001
2003	10	106	55.7	1.98	0.001
2004	12	100	55.1	1.88	0.002

**Table 3:** The mostly likely clusters of TB cases detected using the space-time analysis

Data Years	Cluster Areas(n)	Cluster Years	Observed Cases	Expected Cases	Relative Risk	p-value
1999–2004	13	2000	354	287.8	1.23	0.015
2000–2004	13	2000	354	282.1	1.26	0.001
2001–2004	4	2001	216	178.3	1.21	0.400
2002–2004	1	2002	8	3.1	2.58	0.696
2003–2004	4	2004	20	12.5	1.60	0.568

## Discussion

In this study, two different methods were used to analyze TB cases, using minimal assumptions about the spatiotemporal characteristics of TB clusters. Several notable points can be drawn from the findings. First, there was partial concurrence between the two methods with respect to the results of the analyses. Specifically, the space-time analysis reported an excess incidence of TB in the northern part of Fukuoka in the year 2000 cluster (Fig. 3), and the purely spatial analysis also reported an excess incidence of TB in the same area in 2000 (Fig. 2). This finding and the statistical power of the two analyses strongly suggests that (1) a local cluster, not explainable by chance, existed in the northern area, and (2) a local cluster was detected in 2000.

Second, as mentioned, the two types of analysis identified the most likely cluster in the north of Fukuoka Prefecture in 2000. After 2000, no likely cluster was detected. This result was consistent with national trends [6,32,33]. After 38 years of steady decline, a sudden jump in the number of new cases in Japan was reported in 1997 [6,32,33]. The morbidity rate also increased for the first time in 43 years [6,34,35]. Faced with the resurgence of TB, the Ministry of Health and Welfare declared a state of emergency in 1999. To bring TB under control, the Ministry of Health and Welfare worked together with related public sectors, including local governments and organizations. Strategies included educating the public about the dangers of TB as a re-emergent epidemic; implementing countermeasures, such as medical examinations regulated by the Tuberculosis Prevention Law; monitoring TB carefully; and promoting special anti-tuberculosis programs [6,32,36,37]. Beginning in 2000, the number of TB cases in Japan began to decrease again [6,32,33]. Again, since our finding was in line with a national trend, it was suggested that the decline in cases was consistent with the efforts of the policies implemented in Fukuoka Prefecture.

Third, in the two types of analysis, the most likely clusters consisted of the Chikuho coal mining area (in 1999, 2002, 2003, 2004), Kita-Kyushu industrial area (in 2000), and Fukuoka urban area (in 2001). Backed by the neighboring Chikuho coal mines and harbors, the Kita-Kyushu industrial area originated in 1901. In recent years, however, heavy industry has moved to neighboring countries, where labor is inexpensive. Concomitantly, the economy of the Kita-Kyushu industrial area has deteriorated in the last ten years. Several studies have already reported that the socioeconomic situation in an area influences the incidence of TB in that area [38-47]. Again, since the Kita-Kyushu area was included in the two types of analysis, our finding was consistent with these previous findings.

Fourth, we detected the most likely cluster in the Chikuho coal mining area (in 1999, 2002, 2003, 2004). Several studies have already reported that TB, pneumoconiosis, and COPD, which result from the working conditions in coal mines, were prevalent in the coal mining area [12-17]. Our findings agree with those findings.

We need to mention the practical implications of our findings. If a disease atlas was always complemented with a test for the detection of clusters, public health officials could better prioritize the regions in which thorough investigations should be conducted, while minimizing the time taken to detect genuine abnormalities. To detect disease clusters, the public-health community has historically relied on the watchful eyes of physicians and other health-care workers, who report individual cases, or clusters of cases, of particular diseases to health-care and other authorities. However, the increasing availability of surveillance data raises the possibility of disease cluster detection, and subsequent intervention, if suitable analytic methods are found. To delineate spatial clusters, the space-time scan statistic has the distinct advantage of providing statistical significance, providing a more objective basis for identifying clusters. The method can be an important tool for public health officials and is applicable to the detection of clusters of other diseases.

Our study had several limitations. First, we analyzed a short period of time (i.e., 6 years, from 1999 to 2004). Additional analyses are needed to evaluate the spatial and temporal changes in the pattern of TB using data for a longer study period, or more detailed incidence data (monthly, weekly, or daily). Second, since the study was based on the assumption of circular spatial scanning windows and space-time cylinders, the centroid of each cluster location (i.e., administrative districts such as cities, towns, and villages) is not necessarily included in the cluster circle (Figs. 2 and 3). As a matter of fact, it is almost impossible for an actual administrative area with complicated geographical boundaries to be included in the detected circular cluster. This is another limitation of our methodology. If more advanced methods with more flexible scanning window shapes were used, the centroids of each area might be included in the cluster. Additional methodological research is necessary to relax the circular and cylinder assumptions. Third, the geographical boundary of the detected cluster is not necessarily the boundary of the true cluster. Although we used a circle as the base for the scanning cylinder in this study, other scanning window shapes also exist [28], and circular scan statistics can detect noncircular cluster areas [48]. The sophistication of the analytical methodology, however, is a topic for future study.

## Conclusion

In summary, using data from 1999 to 2004, we used the space-time scan statistic to examine clusters of TB in Fukuoka Prefecture, Japan. The most likely significant cluster for a high occurrence of TB was identified in the north part of Fukuoka Prefecture. Since the efficacy of TB control measures in specific areas could be evaluated by reviewing the control measures and a longitudinal change in TB prevalence, the space-time scan statistic can contribute to health program evaluation. Although the method used here could help prioritize the assignment and investigation of diseases, the applicability of our methodology might be limited. We need to develop more sophisticated analytical methodology for future studies.

## Competing interests

The author(s) declare that they have no competing interests.

## Authors' contributions

DO has made substantial contributions to conception and design, analyzed and interpreted data and drafted the manuscript. AH has been involved in drafting the manuscript or revising it critically for important intellectual content and has given final approval of the version to be published. All authors read and approved the final manuscript.

## Pre-publication history

The pre-publication history for this paper can be accessed here:


